# Engineering cancer stem-like cells from normal human lung epithelial cells

**DOI:** 10.1371/journal.pone.0175147

**Published:** 2017-04-05

**Authors:** Ken Sasai, Etsuko Takao-Rikitsu, Taiko Sukezane, Emmy Yanagita, Harumi Nakagawa, Machiko Itoh-Yagi, Yukina Izumi, Tomoo Itoh, Tsuyoshi Akagi

**Affiliations:** 1 KAN Research Institute, Inc., Kobe, Hyogo, Japan; 2 Division of Diagnostic Pathology, Kobe University Graduate School of Medicine, Kobe, Hyogo, Japan; Università degli Studi della Campania "Luigi Vanvitelli", ITALY

## Abstract

It has been proposed that a subpopulation of tumour cells with stem cell-like characteristics, known as cancer stem cells (CSCs), drives tumour initiation and generates tumour heterogeneity, thus leading to cancer metastasis, recurrence, and drug resistance. Although there has been substantial progress in CSC research into many solid tumour types, an understanding of the biology of CSCs in lung cancer remains elusive, mainly because of their heterogeneous origins and high plasticity. Here, we demonstrate that engineered lung cancer cells derived from normal human airway basal epithelial cells possessed CSC-like characteristics in terms of multilineage differentiation potential and strong tumour-initiating ability. Moreover, we established an *in vitro* 3D culture system that allowed the *in vivo* differentiation process of the CSC-like cells to be recapitulated. This engineered CSC model provides valuable opportunities for studying the biology of CSCs and for exploring and evaluating novel therapeutic approaches and targets in lung CSCs.

## Introduction

Lung cancer is the leading cause of cancer-related mortality, resulting in more than one million deaths worldwide annually [1]. Non-small-cell lung cancer (NSCLC), of which adenocarcinoma is the most common histological subtype, accounts for approximately 80% of all lung cancer cases and is often diagnosed at an advanced, inoperable stage [2]. Even in operable cases, the rate of recurrence is high, and overall 5-year survival rates for NSCLC remain low despite advances in early detection and standard treatment [3]. As in many other cancers, phenotypic and functional heterogeneity among cancer cells within the tumour make curing lung cancer difficult [4]. It is now widely accepted that this heterogeneity is generated mainly by cancer stem cells (CSCs), which have both tumour-initiating ability and differentiation potential [5, 6]. Several studies have reported the isolation and characterization of lung CSCs, and several putative markers for lung CSCs have been identified, including CD133, CD44, CD166, aldehyde dehydrogenase (ALDH) and side population (SP) phenotypes [7–9]. However, controversies and uncertainties remain, and no consensus markers for lung CSCs have yet been identified [7]. This is most likely due to the heterogeneous origins and high plasticity of lung CSCs [8, 9].

Previously, we engineered tumorigenic cells from normal human small airway epithelial cells (HSAECs) via combined expression of multiple defined genetic elements, all of which are known to be highly relevant to lung cancer development. We used different oncogene combinations to generate various types of tumorigenic cells with different histological features and that exhibited varying degrees of differentiation on subcutaneous transplantation into nude mice [10]. Among these cells, tumours formed by HSAEC_4T53RD cells had adenocarcinoma-like histology with glandular structures resembling those observed in clinical lung cancer cases [10]. In the current study, we demonstrate that the HSAEC_4T53RD cells possess characteristics of CSCs in terms of multilineage differentiation potential and strong tumour-initiating ability. Moreover, we establish an *in vitro* 3D culture system that recapitulates the *in vivo* differentiation process of HSAEC_4T53RD cells by which heterogeneous cell populations are generated.

## Materials and methods

### Cells and cell culture

The establishment of HSAEC_4T53RD cells has been described previously [10]. Briefly, normal HSAECs, purchased from Lonza (Walkersville, MD, USA), were malignantly transformed by the introduction of Cdk4, hTERT, a dominant negative p53 mutant, K-rasV12, and cyclin D1, using retroviral vectors. The cells were cultured on collagen-coated dishes in serum-free SAGM medium supplemented with growth factors supplied by the manufacturer (SAGM Bullet Kit; Lonza), and were maintained at 37°C in a low-oxygen environment (3% O_2_ and 5% CO_2_) in a humidified incubator. TIG-3 human lung fibroblasts were obtained from the Japanese Collection of Research Bioresources Cell Bank and were cultured in DMEM supplemented with 10% FBS at 37°C in a low-oxygen environment (3% O_2_ and 5% CO_2_) in a humidified incubator.

### Generation of single-cell-derived HSAEC _4T53RD clones by limiting dilution

HSAEC_4T53RD cells were diluted and plated in 96-well plates at a concentration of 0.1 cells per well. Each well was checked with an inverted microscope several hours after plating, and wells containing a single cell were marked. The colonies in these marked wells were expanded.

### *In vitro* differentiation culture

For *in vitro* differentiation, HSAEC_4T53RD cells were co-cultured with TIG3 human lung fibroblasts in Matrigel (BD Bioscience, CA, USA) as described by McQualter *et al*.[11] with some modifications. Briefly, HSAEC_4T53RD cells (1 x 10^3^ cells) and TIG3 (1 x 10^5^ cells) were mixed and resuspended in 100 μL of Matrigel prediluted 1:1 (vol/vol) with the co-culture medium (DMEM/F-12, GlutaMAX (Thermo Fisher Scientific, MA, USA) supplemented with 10% foetal calf serum, ITS-G (Thermo Fisher Scientific, MA, USA) and antibiotic–antimycotic (Thermo Fisher Scientific, MA, USA)). The cells suspended in Matrigel were then added to a 24-well transwell filter insert (Millicell-CM; Merck Millipore, MA, USA) in a 12-well tissue culture plate containing 1 mL of co-culture medium. Cultures were incubated at 37°C in a low-oxygen environment (3% O2 and 5% CO2) in a humidified incubator for 10–14 days and refed three times per week.

### Sphere immunostaining

After 10–14 days of *in vitro* differentiation culture, spheres were collected using Cell Recovery Solution (BD Bioscience, CA, USA) according to the manufacturer’s protocol, and were then fixed in 2% paraformaldehyde (PFA)/phosphate-buffered saline (PBS) overnight at 4°C. The fixed spheres were treated with 50 mM NH_4_Cl/PBS for 30 min at room temperature to block PFA and were then permeabilised by incubation with 0.2% (vol/vol) Triton X-100/PBS for 30 min at room temperature. The spheres were washed with PBS and blocked with 20% Block Ace (DS Pharma Biomedical, Osaka, Japan) overnight at 4°C, then incubated with primary antibodies overnight at 4°C, washed with PBS, incubated at room temperature for 3 h with appropriate AlexaFluor-conjugated secondary antibodies (Thermo Fisher Scientific, MA, USA), and counterstained with SlowFade Gold Antifade Mountant with DAPI (Thermo Fisher Scientific, MA, USA). The immunostained spheres were analysed using a Nikon A1 confocal microscopy system (Nikon, Tokyo, Japan).

### Xenograft propagation

All the procedures related to animal handling, care, and the treatment in this study were performed according to the guidelines approved by the Institutional Animal Care and Use Committee (IACUC) of KAN Research Institute, Inc.. Single-cell suspensions of 2 x 10^6^ HSAEC_4T53RD cells, unless otherwise indicated, were resuspended in 50% Matrigel and injected subcutaneously in the flank of 6- to 8-week-old female athymic nude mice (BALB/c nu/nu; Japan SLC, Hamamatsu, Japan) or NOD SCID mice (CLEA Japan, Tokyo, Japan). Mice were monitored for overall health status daily and their tumor volumes were measured twice a week using a digital caliper throughout the experiment, and tumour volumes were calculated according to the following formula: ab^2^/2 (a, width; b, length). HSAEC_4T53RD cells resuspended in 50% Matrigel were also implanted under the renal capsule of NOD SCID mice as previously described [12,13]. In order to minimize any suffering during surgical procedures, animals were anesthetized by isoflurane. Tumors were harvested from specified mice. Mice were humanely euthanized by cervical dislocation if their body weights dropped more than 20% of the original weight for two consecutive measurements, their xenograft tumor grew larger than 2000 mm^3^, or they showed apparent moribund at any time. No mortality occurred prior to the end of the study. In all animal studies, food and water were available ad libitum. Animals were housed with no more than 6 per cage in a barrier facility with a high efficiency particulate arrestance (HEPA)-filtered air conditioning under standard 12-hour light/dark cycles.

### Histological analysis and immunohistochemistry

Formalin-fixed, paraffin-embedded xenograft tissues were cut into 4-um sections, and the sections were stained with haematoxylin and eosin, and alcian blue, according to standard protocols. Immunohistochemistry was conducted as described previously [12] using the antibodies listed in S1 Table. For immunofluorescence analysis, tumour-bearing mice were perfused intracardially with 1% paraformaldehyde, and xenograft tumours were removed and postfixed overnight at 4°C. Tumours were then placed into 10% sucrose for 4 h and 20% sucrose overnight, and embedded in optimized-cutting-temperature compound. The embedded tissues were sectioned using a cryostat (Leica Biosystems, Wetzlar, Germany). 10-um-thick sections were used for immunohistochemistry as described previously [13] using the antibodies listed in S1 Table.

## Results

### HSAEC_4T53RD cells generate heterogeneous tumour tissue and have strong tumour-initiating ability

**Fig 1:**
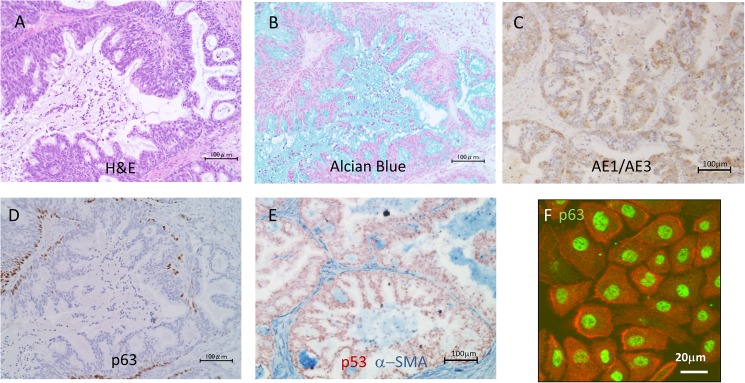
Phenotype of HSAEC_4T53RD xenografts. Formalin-fixed, paraffin-embedded sections (4-μm thick) of xenografts derived from HSAEC_4T53RD cells were subjected to staining with (A) haematoxylin and eosin or (B) alcian blue; or with antibodies against (C) cytokeratins (AE1/AE3), (D) p63, (E) alpha-SMA (blue) and p53 (brown). (F) HSAEC_4T53RD cells cultured *in vitro* on collagen-coated dishes in serum-free SAGM medium were stained with anti-p63 antibody (green) and phalloidin (red).

We previously reported a range of tumorigenic cells engineered from normal HSAECs [10]. These included HSAEC_4T53RD, which express Cdk4, hTERT, a dominant negative mutant of p53, K-rasV12, and cyclin D1. On subcutaneous transplantation into nude mice, these cells formed tumours that showed adenocarcinoma-like histology: they exhibited glandular structures with mucin production, revealed by alcian blue staining (Fig 1A and 1B). These glandular structures consisted of heterogeneous cell types in terms of expression of cytokeratins (Fig 1C) and the basal cell marker p63 (Fig 1D); and were surrounded by stromal cells that stained positive for alpha-SMA and negative for p53, and were therefore supposed to be of mouse origin (Fig 1E). Interestingly, although most HSAEC_4T53RD cells expressed p63 when cultured *in vitro* on collagen-coated dishes in serum-free SAGM medium (Fig 1F), p63-positive cells were confined to the periphery of the glandular structures, adjacent to the surrounding stroma, in xenograft tumours (Fig 1D).

**Fig 2:**
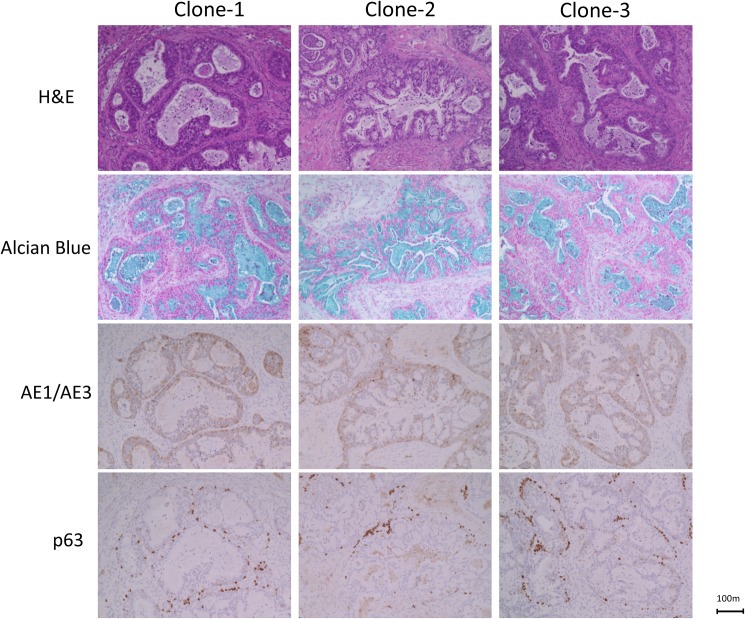
Immunohistochemical analysis of xenografts formed by single-cell-derived HSAEC_ 4T53RD clones. Sections of xenografts formed from three independent single-cell-derived HSAEC_ 4T53RD clones were subjected to haematoxylin and eosin staining (top panels) and alcian blue staining (second panels). Immunohistochemical analyses were also performed using antibodies against cytokeratins (AE1/AE3; third panels) and p63 (bottom panels). Scale bar, 100 μm.

Given that p63-positive basal cells have been identified as stem cells in mouse trachea and human airway epithelium [14], we hypothesized that the heterogeneous tumour histology was generated via differentiation of these p63-positive cells. To investigate this hypothesis, we generated single-cell-derived clones by limiting dilution of parental HSAEC_4T53RD cells. When subcutaneously transplanted into nude mice, six out of seven of these clones formed tumours with adenocarcinoma-like histology containing heterogeneous cell types as did the parental cells, with one exceptional case forming poorly differentiated tumor. Results of histological examination of three representative clones were shown in Fig 2.

**Fig 3:**
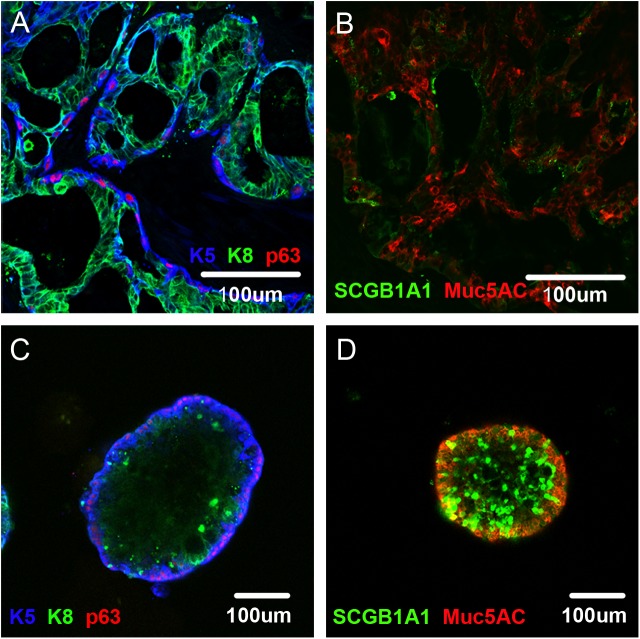
Expression of differentiation markers in both *in vivo* xenografts and *in vitro* differentiation cultures. Sections of xenografts formed from a single-cell-derived HSAEC_4T53RD clone were subjected to staining for (A) K5 (blue), K8 (green) and p63 (red); and (B) SCGB1A1 (green) and MUC5AC (red). Spheres formed by one of the single-cell-derived HSAEC_4T53RD clones were subjected to staining for (C) K5 (blue), K8 (green) and p63 (red); and (D) SCGB1A1 (green) and MUC5AC (red).

Immunofluorescence analysis of the xenograft tumour derived from one of these clones revealed cells expressing the Clara cell marker SCGB1A1, and cells expressing the goblet cell marker MUC5AC [15], in addition to p63-positive basal cells in the tumour tissue (Fig 3A and 3B). Given that both the Clara and goblet cell linages are reported to originate from basal cells [14], these results clearly indicate that the heterogeneity observed in the xenograft tumour tissue resulted from differentiation of single–cell-derived clones with stem cell characteristics.

Moreover, a tumourigenicity assay in NOD/SCID mice revealed that HSAEC_4T53RD cells were able to form tumours with the injection of only 10 cells, albeit with low incidence (1/8), indicating the strong tumour-initiating ability of these cells (Table 1). Taken together, these results demonstrate that HSAEC_4T53RD cells meet the two fundamental criteria for CSCs; that is, they possess multilineage differentiation potential and strong tumour-initiating ability [16].

**Table 1:** Tumour-initiating ability of HSAEC_4T53RD cells.

Number of cells injected	10^4^	10^3^	10^2^	10^1^
Tumour formation incidence	2/2	4/5	3/5	1/8

The indicated numbers of HSAEC_4T53RD cells were injected subcutaneously with 50% Matrigel into NOD/SCID mice, and mice were observed for 10 wks.

### Differentiation of HSAEC_4T53RD cells *in vitro*

**Fig 4:**
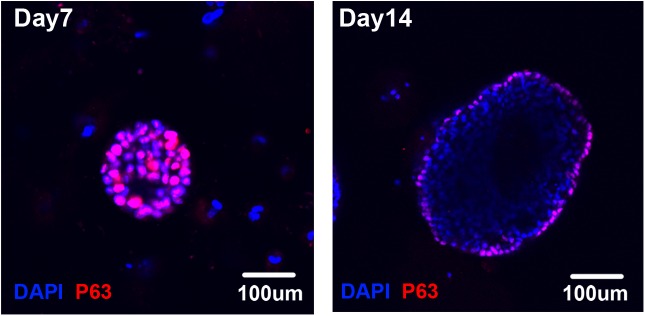
Expression of p63 in *in vitro* differentiation cultures. Spheres formed by one of the single-cell-derived HSAEC_4T53RD clones were subjected to staining with anti-p63 antibody (red) and DAPI (blue) at 7 and 14 days after plating.

To gain more direct evidence for the differentiation of p63-positive cells, we attempted to establish an *in vitro* culture system in which the *in vivo* differentiation process could be recapitulated. To this end, we used a previously reported 3D culture developed for the functional assay of stem cells in the adult mouse lung [11]. Single-cell-derived clonal HSAEC_4T53RD cells were embedded in Matrigel together with TIG3 normal human lung fibroblasts, and the formation of spherical colonies was observed. Although most of the cells in the sphere were p63-positive at day 7 of culture, only the cells in the outer layer were p63-positive when the colonies had grown to several hundred μm in diameter at day 14 (Fig 4).

Immunostaining these spheres with antibodies against keratin 5 (K5) and keratin 8 (K8) revealed that the peripheral p63-positive cells were also positive for K5, as reported previously, whereas the p63-negative cells in the central region were positive for K8 (Fig 3C). This staining pattern was consistent with that of the *in vivo* xenografts (Fig 3A). Moreover, SCGB1A1-positive Clara cells and MUC5AC-positive goblet cells were also present within the sphere colonies, indicating that differentiation into these lineages took place in this 3D culture, as observed in xenografts (Fig 3 B and 3D).

## Discussion

To our knowledge, this is the first report of generation of cells possessing CSC-like characteristics from normal human lung epithelial cells. A number of studies have previously reported the isolation and characterisation of lung CSCs from both established cell lines and clinical specimens [7]. Although several markers, including CD133, CD44, ALDH, and SP, have been used to enrich cell populations for lung CSCs, controversies and uncertainties remain [7]. For example, Meng *et al*. reported that both CD133-positive and CD133-negative populations of lung cancer cell lines contain CSCs [17]. Similar results were also reported for ALDH, and SP phenotypes [18, 19]. Using NSCLC cell lines and patient-derived primary adenocarcinoma cells, Akunuru *et al*. reported that CSCs were enriched in multiple phenotypically distinct subpopulations [20]. Such conflicting results are likely attributable to the heterogeneous origins and high plasticity of lung CSCs [8, 9]. Considering the inconsistencies in the markers identified for lung CSCs, it is preferable to define CSCs based on their biological functions. Here, we demonstrated that HSAEC_4T53RD cells have the potential to differentiate into several lineages and exhibit strong tumour-initiating ability, which are the two most fundamental biological properties of CSCs. Recently, accumulating evidence has indicated the existence of several different types of stem/progenitor cells in normal adult lung, for example airway basal cells [14], variant Clara cells [21], bronchioalveolar stem cells [22], and alveolar epithelial type 2 (AT2) cells [23]. HSAECs, which we used as the starting cell type in this study, are thought to be distal airway basal cells with intrinsic multipotent differentiation capacity [24, 25]. By introducing several defined genetic elements (hTERT, Cdk4, a dominant negative p53 mutant, K-rasV12, and cyclin D1), we conferred the HSAECs with immortality and tumourigenicity without removing their capacity to function as multipotent progenitors. Airway basal cells are generally thought to be the cellular origin of lung squamous cell carcinoma, whereas AT2 cells are the cellular origin of lung adenocarcinoma [26, 27]. More recently, it has been reported that not only AT2 cells, but also Clara cells and bronchioalveolar stem cells, can be the cellular origin of lung adenocarcinoma, depending on conditions, in several genetically engineered mouse models [28–30]. Malkoski *et al*. also reported that adenocarcinoma was more often observed than squamous cell carcinoma when K-rasV12 is specifically expressed in lung basal cells in a genetically engineered mouse lung cancer model [31]. It is now increasingly recognized that basal cells exhibit increased plasticity and multipotency in response to injury, and play pivotal roles as stem cells in the process of tissue regeneration [32], and therefore are at high risk for oncogenic transformation. Moreover, through the analysis of three independent data sets of clinical samples, Fukui *et al*. reported the existence of lung adenocarcinoma subtypes showing unique gene expression features of airway basal cells with aggressive clinical phenotypes, and proposed that airway basal cells are a cellular source of molecular changes associated with the development of a subset of aggressive lung adenocarcinomas in humans [33]. We propose HSAEC_4T53RD cells as a CSC model of such basal cell-derived lung adenocarcinoma.

Although it seems ideal to use CSCs isolated from clinical samples to study CSC biology, there are several problems with this approach. First, as mentioned previously, no definitive markers of lung CSCs have yet been identified, and therefore it is currently technically very challenging to purify CSCs from clinical lung cancer samples [7]. Second, clinical tumour samples harbour many genetic changes, with high variability between patients. Sequencing data from cancer genome projects has revealed approximately 150 mutations per tumour on average in lung cancer [34], and the combinations of mutations differ between patients, resulting in huge diversity. Thus, it is difficult to identify common molecular mechanisms underlying the nature of CSCs against a background of such extensive variation. It is therefore a very powerful approach to engineer surrogate CSC models that can recapitulate CSC biology with the minimum of essential genetic changes necessary to confer CSC properties on normal cells. As we previously reported, the combination of the genetic changes used in the current study is minimally sufficient to induce tumorigenic transformation in HSAECs [10], and all of which are well recognized to have considerable clinical relevance. For example, more than 85% of lung adenocarcinoma samples were positive for the expression of hTERT [35]. According to the genome analysis of 230 resected lung adenocarcinomas by the Cancer Genome Atlas Research Network, the rate of the loss of p53 function was 63%, and of the inactivation of the RB pathway (such as by the homozygous deletion of *CDKN2A* and by the amplification of *CDK4* and/or *CCND1*) was 64% [36]. *KRAS* is the most frequently activated oncogene in lung adenocarcinoma, and was mutated in 32% of cases [36]. In contrast to other driver oncogenes in lung cancer, such as *EGFR* and *ALK*, molecularly targeted drugs to *KRAS* have not yet been developed and therapeutic targeting of *KRAS*-mutated lung adenocarcinoma remains a huge challenge. Despite the lack of reports characterizing the human lung cancer stem cells in relation to the mutated oncogenes so far, it has been demonstrated in genetically engineered mouse lung adenocarcinoma model that the biological properties of cancer stem cells differ significantly depending on the driver oncogenes [37].

The engineered CSC model described in this study represents a valuable experimental system for the study of basic CSC biology of *KRAS*-mutated lung adenocarcinoma, including the molecular mechanisms governing self-renewal and differentiation; and also should greatly contribute to the discovery and evaluation of novel therapeutic approaches and targets in CSCs of this intractable lung cancer.

## Supporting information

S1 Table Antibody information. Antibodies used in this study are listed with the clone names (if applicable), suppliers and dilution conditions.
(DOCX)
