# Supplementary material for: Engineering cancer stem-like cells from normal human lung epithelial cells
Source: PLoS One. 2017 Apr 5;12(4):e0175147. doi: 10.1371/journal.pone.0175147 (PMC5381922; doi:10.1371/journal.pone.0175147)
Supplement: S1 Table — Antibodies used in this study are listed with the clone names (if applicable), suppliers and dilution conditions. (DOCX) [file pone.0175147.s001.docx]

**Supporting information**

**S1 Table. Antibody information**

Antibodies used in this study are listed with the clone names (if applicable), suppliers and dilution conditions.

| Antibody | | Supplier | Dilution |
| --- | --- | --- | --- |
| Immunohistochemichal Analyses (FFPE tissue) | |  |  |
|  | Anti-multi-cytokertatins (AE1/AE3) | Dako (Glostrup, Denmark) |  |
|  | Anti-p53 (DO7; mouse monoclonal) | Novocastra (Newcastle, UK) | 1:300 |
|  | Anti-p63 (4A4; mouse monoclonal) | Dako (Glostrup, Denmark) | 1:100 |
|  | Anti-alpha SMA (1A4; mouse monoclonal) | Nichirei (Tokyo, Japan) |  |
| Immunohistochemichal Analyses (Frozen tissue) | |  |  |
|  | Anti-p63 (4A4; mouse monoclonal) | Santa Cruz (Santa Cruz, CA, USA) | 1 :20 |
|  | Anti-MUC5AC (CLH2; mouse monoclonal) | Santa Cruz (Santa Cruz, CA, USA) | 1 :200 |
|  | Anti-KRT-5 (Poly19055; rabbit polyclonal) | BioLegend (San Diego, CA, USA) | 1 :200 |
|  | Anti-KRT-8 (TROMA-I-5; rat monoclonal) | Developmental Studies Hybridoma Bank (Iowa City, IA, USA) | 1 :50 |
|  | Anti-SCGB1A1 (394324 ; mouse monoclonal) | LifeSpan BioSciences (Seattle, WA, USA) | 1 :100 |
